# Effects of a Proteinase Inhibitor from *Inga laurina* Seeds (ILTI) on *Aedes aegypti* Larval Development

**DOI:** 10.3390/jox15030077

**Published:** 2025-05-22

**Authors:** Ana Jacobowski, Welington Leite, Antolim Martinez Júnior, Eduarda Reis, Lorena Pires, Vitória Silva, Layza Rocha, Eduardo Arruda, Octávio Franco, Marlon Cardoso, Priscila Hiane, Maria Macedo

**Affiliations:** 1 Laboratory of Protein Purification and Their Biological Functions, Federal University of Mato Grosso do Sul, Campo Grande 79.050-010, MS, Brazil; anacristinaj@gmail.com (A.J.); farmwelington@gmail.com (W.L.); antolim.penha@gmail.com (A.M.J.); eduarda.thiburcio@ufms.br (E.R.); lorena.pires@ufms.br (L.P.); vitoriaf.alunoufms@yahoo.com (V.S.); layzasarocha@gmail.com (L.R.); priscila.hiane@ufms.br (P.H.); 2 TechnoAnlogical Chemistry Laboratory, Federal University of Grande Dourados, Dourados 79.825-070, MS, Brazil; ejarruda@gmail.com; 3 S-Inova Biotech, Postgraduate Program in Biotechnology, Don Bosco Catholic University, Campo Grande 79.117-900, MS, Brazil; ocfranco@gmail.com (O.F.); marlonhenrique6@gmail.com (M.C.); 4 Postgraduate Program in Environmental Sciences and Agricultural Sustainability, Don Bosco Catholic University, Campo Grande 79.117-900, MS, Brazil

**Keywords:** *Aedes aegypti*, digestive enzyme, insecticide, Kunitz-type inhibitor, molecular docking, protease

## Abstract

*Aedes aegypti* (Linnaeus, 1762) is Brazil’s primary vector of epidemiologically significant arboviruses such as yellow fever, dengue, Zika, and chikungunya. Despite using conventional chemical control measures, this species has developed resistance to standard chemical insecticides, prompting the search for natural larvicidal compounds. Plant protease inhibitors offer an insecticidal alternative as the primary digestive enzymes in the midgut of *Ae. aegypti* are proteases (trypsin and chymotrypsin). *Ae. aegypti* larvae fed with ILTI, a Kunitz-type trypsin inhibitor derived from *Inga laurina* seeds, at concentrations between 0.03 mg of protein per mL (mgP/mL) and 0.12 mgP/mL, exhibited delayed larval development, with a lethal concentration (LC_50_) of 0.095 mgP mL^−1^ of ILTI for 50% of fourth-instar larvae (L_4_). The ex vivo assay indicated that ILTI effectively inhibited the activity of larval trypsin, which remained susceptible to the inhibitor. Additionally, molecular modelling and docking studies were conducted to predict the three-dimensional ILTI/enzyme molecular complexes at the atomic level. Therefore, the results demonstrate that ILTI functions as a protease inhibitor in this species, presenting itself as a promising larvicidal tool in the control of *Ae. aegypti*.

## 1. Introduction

*Aedes aegypti* (Linnaeus, 1762) is the primary vector of epidemiologically significant arboviruses, including yellow fever, dengue, Zika, and chikungunya in Brazil. Despite the application of conventional chemical control measures, diseases transmitted by haematophagous arthropod vectors affect approximately 80% of the global population at some point in their lives, according to the World Health Organization (WHO) [1]. Several social, demographic, and environmental factors influence the transmission dynamics of these diseases, including geographic spread, re-emergence, and seasonality. Vector control efforts encompass entomological surveillance and the monitoring of insecticide resistance [2]. Among vectors, the *Ae. aegypti* is one of the main targets for transmitting important arboviruses to humans. The adaptive capacity of *Ae. aegypti* to different environments allows it to survive at higher temperatures and in areas with low rainfall [3]. Control of *Ae. aegypti* has been carried out mainly through conventional chemical insecticides, contributing to the global reduction of the vector population. However, an increase in mosquito resistance to insecticides has been observed [4,5], highlighting the need to adopt alternative methods to combat these vectors.

In this context, larvicides have received greater emphasis, since *Ae. aegypti* larvae are more resistant and have higher concentrations of protease enzymes in their digestive tract [6]. Plant larvicides in aqueous formulations are of paramount importance due to their effectiveness in the most vulnerable phases of *Ae. aegypti* (eggs, larvae, and pupae). By targeting intervention at these stages, larvicides offer a crucial preventive approach that can halt mosquito development before it reaches adulthood; at this point, it becomes a disease vector. This strategy is particularly advantageous compared to insecticides that target adult mosquitoes, as population reduction in the larval stage can lead to more efficient and sustainable control of *Ae. aegypti* [7].

Plant-derived protease inhibitors (PIs) are essential for plant survival and play a crucial role as defense proteins with significant insecticidal activity. PIs act by blocking proteolytic enzymes in insects, directly interfering with the digestive processes of these organisms [8]. This mechanism of action compromises the growth and viability of insects, because proteolytic digestion is essential for their nutrition and development. However, insects can develop resistance to PI by inactivating these substances [9]. This resistance process may involve a variety of mechanisms, including genetic mutations that alter the structure of proteases or differential regulation of gene expression to mitigate the adverse effects of PI. These adaptations allow insects to survive and reproduce even with these inhibitory agents [10].

Mosquito larvae, especially *Ae. aegypti*, present a variety of digestive proteases, with serine proteases, such as trypsin and chymotrypsin, the main ones responsible for proteolytic digestion in the larval and adult stages. These enzymes are essential in breaking down proteins into smaller peptides and amino acids, providing nutrients necessary for the mosquito’s development and survival [11]. The genomic analysis of *Ae. aegypti* revealed the presence of 66 trypsin genes in both larvae and adults, demonstrating a remarkable diversity and abundance of digestive enzymes, such as trypsin, throughout the different life stages of this mosquito [12].

Our research group isolated and characterized a trypsin inhibitor, identified as ILTI (*Inga laurina* Trypsin Inhibitor), from the seeds of *Inga laurina*, a species belonging to the Fabaceae (Leguminosae) family [13]. ILTI, with its 180 amino acid residues arranged into a single peptide chain, exhibits homology with the family of Kunitz-type inhibitors and significant similarity with seed storage proteins, including sprain and miraculin. Previously, we showed that ILTI inhibited bovine trypsin with an equilibrium dissociation constant (Ki) of 6 × 10^−9^ M but did not inhibit bovine chymotrypsin [13]. This inhibitor demonstrated several biological activities, including antimicrobial, insecticidal, antitumor and antibiofilm action, showcasing its multifunctional nature and potential for various applications.

In order to ensure the implementation of new insecticidal protease inhibitors, this study aimed to understand better the mechanisms of action of ILTI in the larval development of *Ae. aegypti* through enzymatic mechanisms of action. Additionally, in silico studies were conducted to predict the three-dimensional arrangement for ILTI and *Ae. aegypti* trypsin, and chymotrypsin. Additionally, molecular docking studies were carried out to predict the interactions and binding affinity values for the ILTI/trypsin and ILTI/chymotrypsin molecular complexes, thus creating a parallel between in vivo and in silico studies.

## 2. Materials and Methods

### 2.1. Chemicals Reagents

All chemical reagents and molecular weight markers used in this research were purchased from Sigma-Aldrich—São Paulo, Brazil.

### 2.2. ILTI Purification

ILTI was isolated according to Macedo et al. (2007) [13]. Briefly, *I. laurina* seeds free of seed coat and defatted with hexane were ground and extracted with potassium phosphate buffer (0.1 M, pH 7.6, 1:10 *w*/*v*) at 4 °C for 4 h under constant stirring. The crude extract was centrifuged (10,000× *g* at 4 °C for 30 min), and the supernatant was collected, dialyzed, and lyophilized. The lyophilized extract was purified in DEAE-Sepharose with Tris-HCl buffer (0.05 M at pH 8), and the chromatographic profile was traced by reading the fractions at 280 nm. The inhibitory activity was identified by antitryptic assay, using bovine trypsin and Nα-Benzoyl-L-arginine-4-nitroanilide hydrochloride (BApNA). The fractions with antitryptic activity were dialyzed, lyophilized, and used in biological assays.

### 2.3. Protein Quantification

The total soluble proteins were quantified according to Bradford’s method [14], using the standard curve of bovine serum albumin (BSA) 1.0 mg of protein per milliliter (1 mgP mL^−1^).

### 2.4. Ae. aegypti Larvae

*Ae. aegypti* eggs (Rockefeller strain) were obtained from the laboratory colony from Oswaldo Cruz Foundation (FIOCRUZ) in Rio de Janeiro, RJ, Brazil. At the LPPFB, eggs were hatched in dechlorinated water. The larvae were fed cat food (Whiskas^®^, Campinas – SP, Brazil) ad libitum and maintained at 26–27 °C, with 80% relative humidity, on a 12 h light:12 h dark cycle [15]. Assays were performed with larvae in instars 1–4, pupae and adults. Once the L4s progressed to the pupal stage, they were placed in screened cages for complete development and emergence into the adult stage.

### 2.5. Effect of ILTI on Larval Development

The larvicidal activity assay was carried out according to the method recommended by the World Health Organization [16] and modified by Coelho et al. [17]. The effects of ILTI on *Ae. aegypti* larval development were evaluated by placing 25 larvae in disposable plastic cups containing 20 mL of distilled water (control group) or distilled water with ILTI (0.03 mgP mL^−1^, 0.06 mgP mL^−1^, 0.09 mgP mL^−1^ and 0.12 mgP mL^−1^). Every 48 h (until 144 h), the surviving larvae were weighed and evaluated for their developmental instar. Larvae that did not respond to touch stimulation with tweezers were considered dead. The average larval weight was determined on an analytical scale by weighing all larvae in the group and dividing the value by the total number of individuals. Food was provided ad libitum daily (1 mg of Whiskas^®^ food/group/day). All experiments were run in triplicate at least.

### 2.6. Midgut Larvae

For midgut dissection, *Ae. aegypti* larvae were immobilised by exposure to low temperatures. Each larval midgut was then carefully excised under chilled conditions to minimise degradation and proteolytic activity. The isolated midguts were homogenised in an ice-cold buffer solution containing 0.05 M Tris-HCl at pH 8.0 to ensure optimal enzyme stability and maintain the physiological pH necessary for subsequent assays. Following homogenisation, samples were centrifuged at 6400× *g* for 10 min at 4 °C to separate cellular debris from the soluble enzyme fraction. The resulting supernatants containing the target enzymatic proteins were promptly aliquoted and stored at −20 °C to preserve enzymatic activity until further analysis.

### 2.7. Enzymatic Activity Assays

Enzymatic assays were conducted using *Aedes aegypti* larvae from the L1 to L4 stages, following World Health Organization guidelines [16]. For each experimental group, 25 larvae were placed in 20 mL of distilled water as a control or exposed to varying concentrations of ILTI, ranging from 0.03 mgP mL^−1^ to 0.12 mgP mL^−1^. This exposure was maintained for 48 h, after which surviving larvae were collected for enzymatic analysis.

The midguts of the surviving larvae were carefully dissected, pooled, and homogenised in an ice-cold Tris-HCl buffer (50 mM, pH 8.0) to stabilize the enzymatic proteins. The homogenates were then centrifuged at 6400× *g* for 10 min at 4 °C to separate the supernatant containing the soluble enzyme fractions, which was subsequently used in the enzymatic assays.

#### 2.7.1. Tryptic Activity

Quantification of residual trypsin activity in the midgut was performed on a microplate according to Erlanger et al. [18]. For this, 2 µg of proteins from the supernatant portion of the larval midgut centrifuge were incubated for 30 min at 37 °C in Tris buffer (50 mM at pH 8.0) with 100 µL of 1 mM BApNA. The resultant enzymatic activity was quantified by measuring the rate of BApNA hydrolysis, monitored at an absorbance of 410 nm, and expressed as nanomoles of BApNA hydrolysed per minute.

#### 2.7.2. Chymotryptic Activity

Quantification of residual chymotryptic activity was performed according to Christeller et al. [19]. For this, 2 µg of proteins from the supernatant portion of the larval midgut centrifuge were incubated for 30 min at 37 °C in Tris buffer (50 mM at pH 8.0), with 100 µL of 1 mM N-Succinyl-Alanine-Alanine-Proline-Phenylalanine-p-nitroanilide (SAApNA). Enzymatic activity was determined in nM of SAApNA hydrolysed per minute, with absorbance measured at 410 nm.

#### 2.7.3. Acetylcholinesterase Activity

According to Ellman et al. [20], systemic acetylcholinesterase activity was evaluated in L_3_ and L_4_ treated with ILTI (0.03, 0.06, 0.09 and 0.12 mgP mL^−1^) and control. For this assay, 40 µg of larval homogenate proteins were incubated (at 25 °C for 5 min) with 25 µL of acetylthiocholine (12.5 mM) and 25 µL of dithionitrobenzoic acid (DTNB—10 mM). The enzymatic reaction was monitored at 409 nm and enzymatic activity was determined in nM of DTNB hydrolyzed per minute.

#### 2.7.4. Acid and Alkaline Phosphatases

The determination of the activity of acid and alkaline phosphatases was carried out according to Asakura [21]. The activity of acid phosphatases was measured by adding 50 µL of larval homogenate to 450 µL sodium acetate buffer (50 mM at pH 4.6). For alkaline phosphatases activity, 20 µL of the larval homogenate was diluted in 500 µL Tris buffer (pH 8.0). The two-reaction media were incubated (at 37 °C for 15 min) with 500 µL of the respective buffer containing 12.5 mM p-nitrophenylphosphate (pNPP), a common substrate for these enzymes. The reaction was stopped by adding NaOH 0.5 M and centrifuged (at 4000× *g* for 5 min). The absorbance of the supernatant was monitored at 440 nm.

### 2.8. Ex Vivo Inhibition of Tryptic Activity in Aedes aegypti L_4_ Exposed to ILTI

The tryptic activity of the midguts of *Ae. aegypti* larvae (L_4_) was quantified as previously described (Section 2.7.1), with modifications to assess the ex vivo response to inhibitors. The larvae were maintained for 48 h on a control diet or supplemented with 0.06 mg mL^−1^ of ILTI. After this period, the midguts were dissected, homogenized in 50 mM Tris buffer (pH 8.0), and centrifuged at 6000× *g* for 10 min at 4 °C. The supernatant containing soluble proteins was collected for analysis. For the inhibition assay, 5 µg of midgut protein was preincubated for 15 min at 37 °C with: (I) 100 mM TLCK, (II) 1 mM PMSF, or (III) 1 mg mL^−1^ IL. A positive control (no inhibitor) was included. After preincubation, 100 µL of 1 mM BApNA was added to each reaction. BApNA hydrolysis was monitored at 410 nm for 30 min at 37 °C in a microplate reader. The residual tryptic activity was expressed as nanomoles of BApNA hydrolyzed per minute (nmol min^−1^).

### 2.9. Molecular Modelling

For molecular modelling studies, the primary sequences of ILTI, trypsin and chymotrypsin from *Ae. aegypti* were obtained from the National Center for Biotechnology Information (NCBI) gene bank. Signal peptide and transmembrane regions were predicted by Phobius server [22] and disregarded for further analyses. Molecular modelling was carried out using Alphafold2 [23]. The PDBsum [24] and Molbrobity [25] servers were used to calculate the structural statistics for the lowest free-energy theoretical models (ILTI, trypsin and chymotrypsin) considering their stereochemistry, geometry, and energy distributions, as well as calculating their overall quality scores in comparison with those scores obtained for proteins already resolved by X-ray crystallography or Nuclear Magnetic Resonance (NMR) techniques.

### 2.10. Molecular Docking

After molecular modelling, the lowest free-energy theoretical models for ILTI, trypsin and chymotrypsin were used for molecular docking simulations, in order to better understand the possible interactions that occur between these inhibitor/protease complexes. AutoDock Tools [26] was used to set up a grid box with 60 × 60 × 60 points and 1 Å spacing located at the centre of both serine proteases. All non-polar hydrogens were added to trypsin and chymotrypsin. Furthermore, AutoDock Tools (1.5.6 version) were also used for protein manipulation, where the maximum freedom to ILTI (ligand) side chains was locked to minimize the computational simulation time. AutoDock 4.2 [26] was used to program 50 runs of molecular docking simulations, with the complexes being ranked according to their affinity values in kcal mol^−1^. Further analyses were carried out by using PyMol (http://www.pymol.org), where all the figures were built and the possible interactions between inhibitor/proteases were predicted, respecting the maximum distance of 4 Å for all atoms involved.

### 2.11. Statistical Analysis

The results obtained were statistically valid through comparisons of means using the Tukey test, with a significance level of 5% (GraphPad Prism v. 9.5.1). The lethal concentration for 50% of the population (LC_50_) was calculated using the Probit method with 95% confidence, using the StatPlus^®^ 2009 program for Windows (AnalystSoft—Vancouver, Canada).

## 3. Results

### 3.1. Effect of ILTI on Ae. aegypti Larval Development

**Figure 1:**
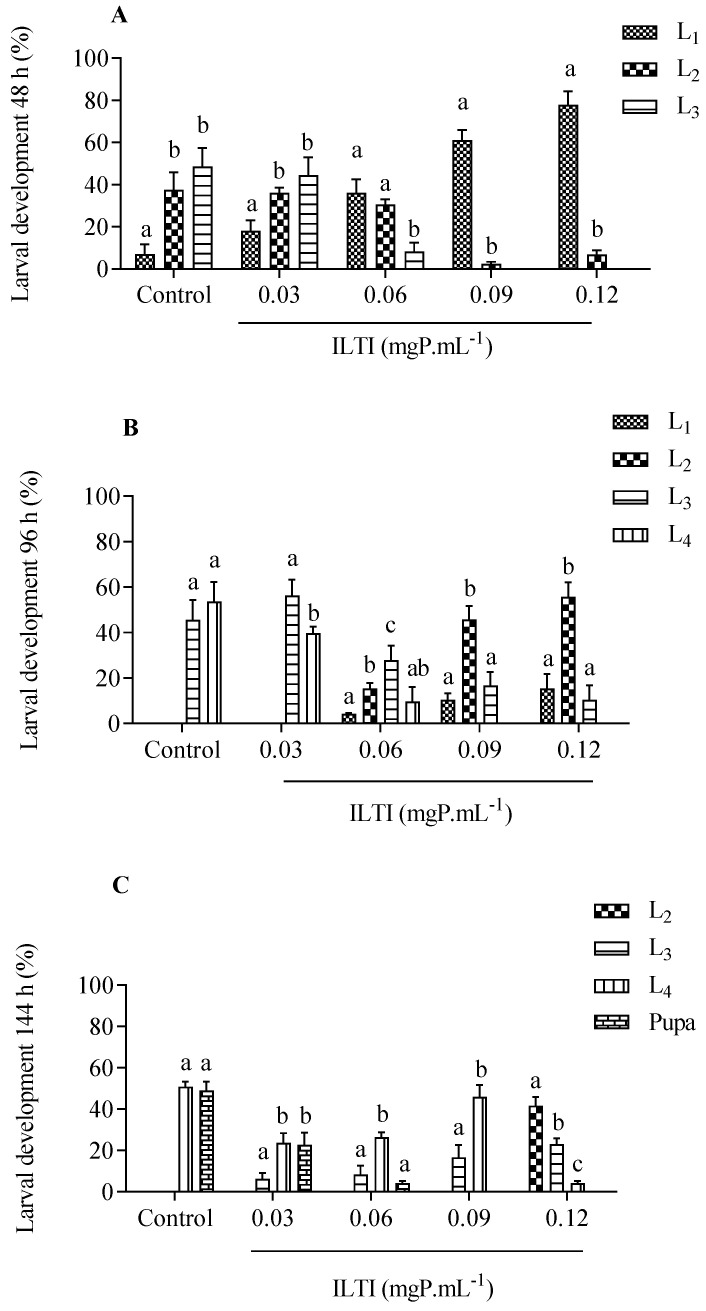
Chronic effect of different concentrations of ILTI on the ongoing development of *Ae. aegypti* larvae exposed to distilled water (control) or ILTI (0.03, 0.06, 0.09 and 0.12 mgP mL^−1^) after 48 h (**A**), 96 h (**B**) and 144 h (**C**). Different letters indicate a significant difference between larvae from the same treatment (ANOVA, *p* < 0.05 followed by Turkey’s post-test). L_1_ (1st larval instar); L_2_ (2nd larval instar); L_3_ (3rd larval instar); L_4_ (4th larval instar).

The biological effect of exposing neonatal *Ae. aegypti* larvae to ILTI (0.03, 0.06, 0.09 and 0.12 mgP mL^−1^) or water (control group) was verified after 48 h, 96 h, and 144 h (Figure 1). After 48 h of treatment, 40% of L_2_ and 50% of L_3_ were observed in the control group. However, larvae treated with higher concentrations of ILTI remained in the initial instars, as occurred in the group that received ILTI 0.09 mgP mL^−1^ (60% L_1_) and in the group 0.12 mgP mL^−1^ (more than 80% L_1_). After 96 h, larvae in the control group reached the third and fourth instars (49% L_3_ and 58% L_4_). The 0.03 mgP mL^−1^ group showed 60% L_3_ and 40% L_4_, whereas the 0.06 mgP mL^−1^ group showed 30% L_3_. Larvae from the groups treated with 0.09 and 0.12 mgP mL^−1^ did not evolve to L_4_ after 96 h, with the majority (45% and 55%, respectively) remaining L_2_.

At the end of treatment (144h), the control group presented 47.2% in the pupa stage and 52.8% in L_4_. The 0.03 mgP mL^−1^ group still presented L_2_ (<10%), a trend observed in other ILTI treatments. The group treated with 0.12 mgP mL^−1^ presented 45% L_2_ and a small number of L_4_.

### 3.2. Effects of ILTI on Ae. aegypti Larval Weight

**Figure 2:**
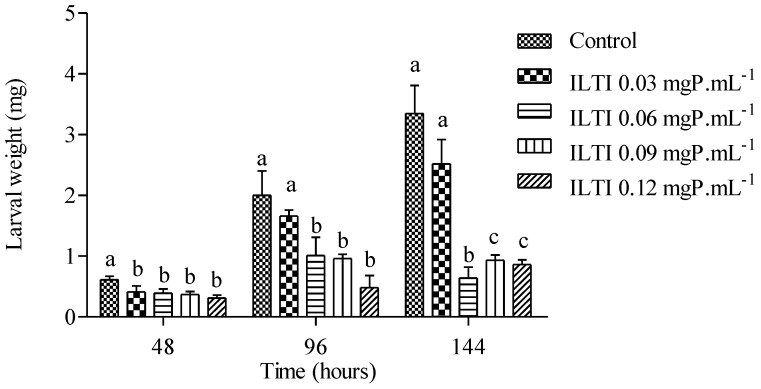
Biological effect of ILTI (0.03, 0.06, 0.09 and 0.12 mgP mL^−1^) on *Ae. aegypti* larval weight after 48 h, 96 h and 144 h of treatment. Bars indicate mean ± SD. Different letters indicate a significant difference (*p* < 0.05) between larvae from the same treatment time (ANOVA, *p* < 0.05).

The effects on the weight of larvae exposed to ILTI are shown in Figure 2.

After 48 h, it is noted that the larvae in the control group had a greater weight than those treated with ILTI, regardless of the concentration. At 96 h of treatment, the difference in weight is not significant between the control and ILTI 0.03 mgP mL^−1^ groups. However, it is more pronounced when compared to the other groups. This allows us to infer that the higher the ILTI concentration supplied to the insect, the greater the difficulty it will have in increasing its weight. After 144 h, larvae in the control group showed average values above 3.0 mg, whereas groups fed with 0.09 mgP mL^−1^ and 0.12 mgP mL^−1^ showed values below 1.0 mg. The control group showed a weight gain of approximately 550% (0.612 mg ± 0.06 to 3.35 mg ± 0.46), whereas the ILTI 0.12 mgP mL^−1^ group had a gain of 220% (0.377 mg ± 0.05 to 0.86 mg ± 0.08).

### 3.3. Effects of ILTI on Ae. aegypti Larval Survival

**Figure 3:**
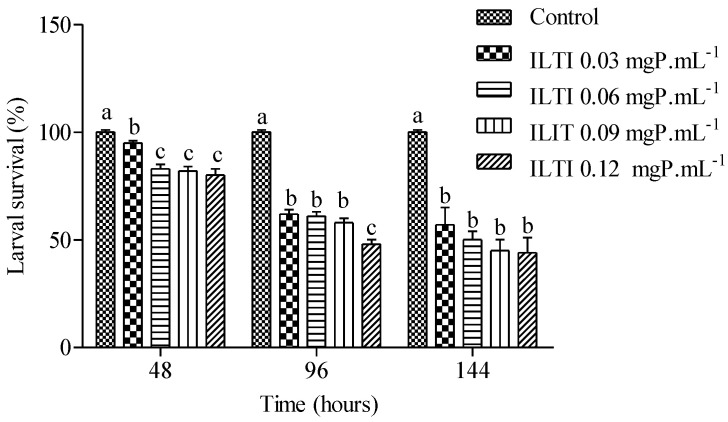
Biological effect of ILTI (0.03, 0.06, 0.09, and 0.12 mgP mL^−1^) on larval survival (%) of *Ae. aegypti* L_4_ after 48 h, 96 h, and 144 h of treatment. Bars indicate mean ± SD. Different letters indicate a significant difference (*p* < 0.05) between larvae from the same treatment time (ANOVA, *p* < 0.05).

The effect of ILTI on the survival of *Ae. aegypti* larvae exposed to increasing ILTI concentrations is demonstrated in Figure 3. An increase in mortality of L_4_ treated with the trypsin inhibitor is noted after 48 h of treatment. Concentrations of 0.03 mgP mL^−1^ or greater were significantly different from the control group. Survival after 96 h of treatment was less than 60% for all groups treated with ILTI, with a reduction after 144 h of treatment. The lethal concentration value for 50% of individuals (LC_50_) for L4 treated with ILTI for 96 h was 0.0954 mgP mL^−1^.

### 3.4. Effect of ILTI on the Tryptic and Chymotryptic Activities of Ae. aegypti

**Figure 4:**
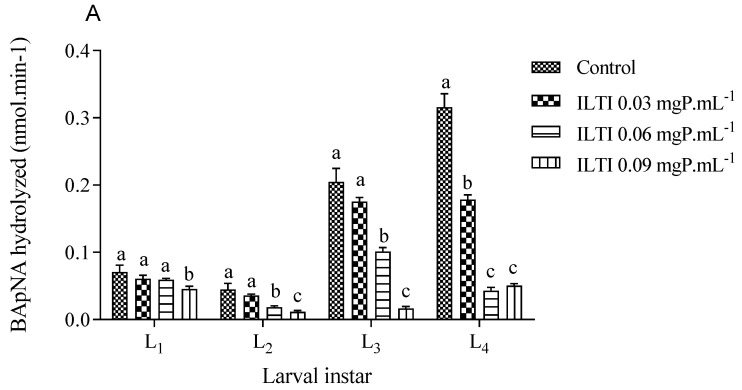

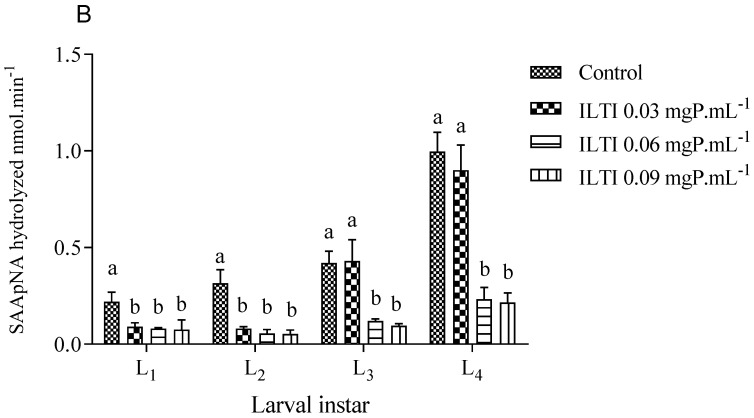
The biological effect of ILTI (0.03, 0.06, and 0.09 mgP mL^−1^) on the residual activity of trypsin and chymotrypsin of *Ae. aegypti* larvae, expressed in the amount of substrate BApNA (**A**) and SAApNA (**B**) hydrolysed per minute. Different letters indicate a significant difference (*p* < 0.05) between larvae of the same instar.

Figure 4 illustrates the residual tryptic and chymotryptic activities of midgut enzymes in larvae treated with ILTI. The treated larvae exhibit a reduction in residual activity across both enzyme groups. Larvae exposed to 0.06 and 0.09 mgP mL^−1^ of ILTI demonstrated up to an 80% decrease in enzymatic activity during the third and fourth instars, compared with the control group.

### 3.5. Effect of ILTI on the Acetylcholinesterase Activity of Ae. aegypti

**Figure 5:**
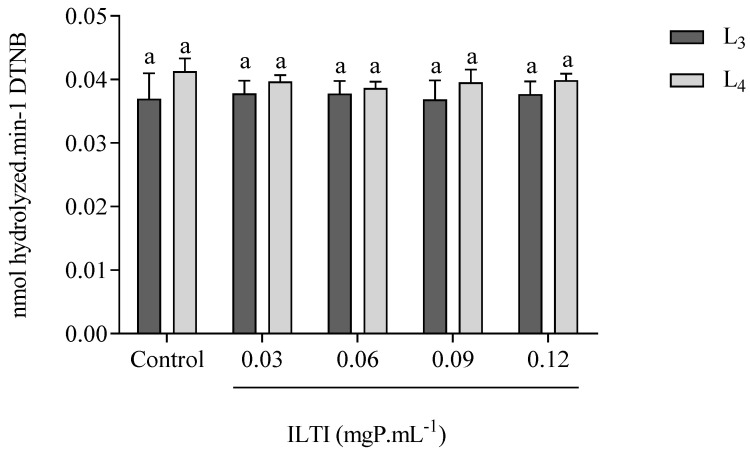
Effect of ILTI on acetylcholinesterase activity in *Ae. aegypti* larvae L_3_ and L_4_, respectively, treated with distilled water (control) and ILTI (0.03, 0.06, 0.09 and 0.12 mgP mL^−1^). Bars indicate residual enzymatic activity, expressed in nmol of dithionitrobenzoic acid released per minute. Equal letters indicate no significant difference (ANOVA, *p* < 0.05) when compared to controls.

The effects of ILTI on the systemic acetylcholinesterase activity of L_3_ and L_4_ of *Ae. aegypti* are demonstrated in Figure 5. It is noted that L_3_ and L_4_ did not show a decrease in the activity of this enzyme when treated with ILTI at the concentrations tested, indicating that this is not a preferential site of the inhibitor.

### 3.6. Effect of ILTI on the Activity of Acid and Alkaline Phosphatases in Ae. aegypti

**Figure 6:**
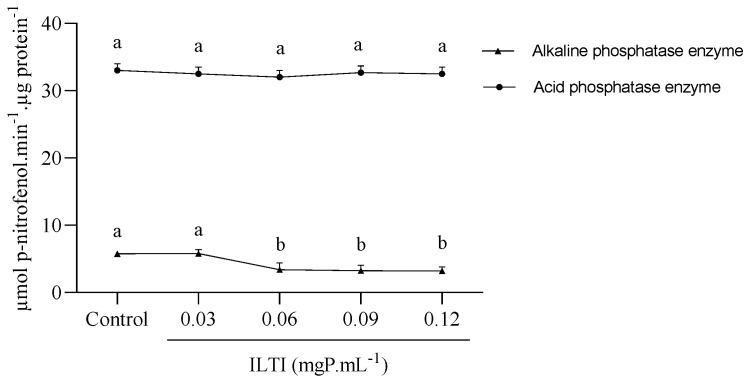
Effect of ILTI on the acid and alkaline phosphatase enzymes of *Ae. aegypti*. The fourth instar larvae remained in contact with distilled water (control) or ILTI (0.03, 0.06, 0.09 and 0.12 mgP mL^−1^) for 48 h. The results indicate the activity of phosphatases, measured in µmol of p-nitrophenol per µg of protein released per minute. Different letters indicate a significant difference (*p* < 0.05) between groups of the same enzyme class.

Figure 6 presents the data on the effects of ILTI on the activity of *Ae. aegypti* L_4_ phosphatases. While the effect of ILTI on acid phosphatases is not visually discernible, the concentration of 0.06 mgP mL^−1^ led to a decrease in the activity of alkaline phosphatases. This reduction could potentially lead to a loss of metabolic functions in these insects, highlighting the importance of our findings.

### 3.7. Ex Vivo Inhibition of Tryptic Activity in Aedes aegypti L_4_ Exposed to ILTI

**Figure 7:**
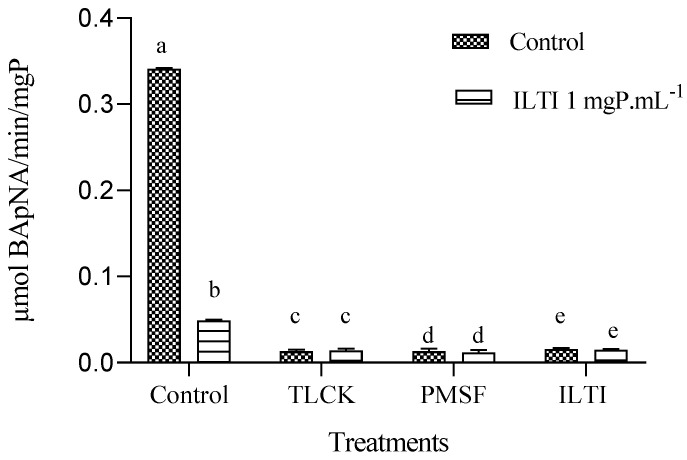
Ex vivo inhibition of trypsin activity in L_4_ midguts from ILTI-fed larvae (0.06 mgP mL^−1^ diet) by serine protease inhibitors (100 mM TLCK, 1 mM PMSF, or 1 mgP mL^−1^ ILTI). Different letters indicate significant differences between treatments within each dietary group (ANOVA/ Tukey, *p* < 0.05).

Midgut extracts from L_4_ larvae fed ILTI (0.06 mgP mL^−1^) showed significant inhibition of residual tryptic activity after ex vivo incubation with serine protease inhibitors (TLCK, PMSF, or ILTI) compared to the inhibitor-free control (Figure 7).

Among the inhibitors tested, ILTI (1 mgP mL^−1^, in vitro) had the strongest effect, reducing enzyme activity by 88.4% relative to the control, followed by TLCK (100 mM) and PMSF (1 mM). No significant difference in inhibitor sensitivity was observed between larvae fed the control diet and those pre-exposed to ILTI (*p* > 0.05), suggesting that dietary ILTI exposure did not alter trypsin response to ex vivo inhibition.

### 3.8. Molecular Modelling and Docking Simulations

After the prediction and removal of signal peptide and intermembrane regions using the phobius server, the mature sequences for ILTI (gi: AFG28551), trypsin (gi: 157137123) and chymotrypsin (gi: 1336053) from *Ae. aegypti* were used for molecular modelling studies using the AlphaFold 2 server. In terms of structural statistics, all the lowest free-energy theoretical models generated revealed >90% of their residues in the most favoured regions of the Ramachandran Plot (Table 1).

Additionally, low percentages of Ramachandran outliers, bad bonds, bad angles and poor rotamers were found for the generated models. By contrast, favoured rotamers presented percentages >98% in all cases. Moreover, the calculated average scores for the dihedral angles associated with the backbone covalent forces (also known as G-factors) were of −0.31, −0.36 and −0.31 for ILTI, trypsin and chymotrypsin, respectively.

**Figure 8:**
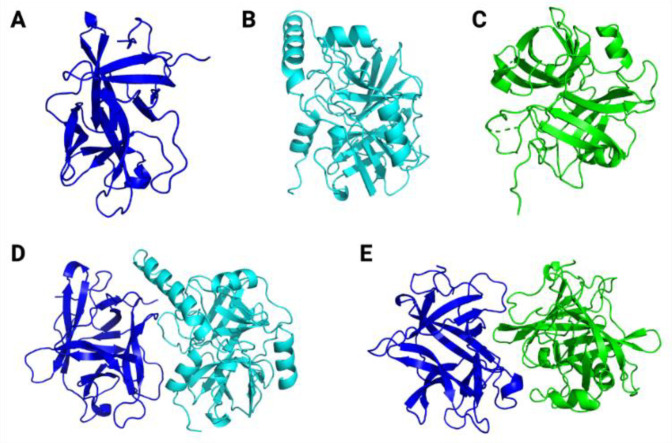
Three-dimensional theoretical models for ILTI (**A**), *Ae. aegypti* trypsin (**B**) and *Ae. aegypti* chymotrypsin (**C**). The predicted conformations for ILTI/trypsin (**D**) and ILTI/ chymotrypsin (**E**) complexes are also represented.

Altogether, these structural statistics indicate the reliability of the theoretical models predicted in the present study. When it comes to overall structural scaffold, ILTI presents β-strands connected by loops (Figure 8A), as commonly reported for other Kunitz-type inhibitors [12]. For trypsin (Figure 8B) and chymotrypsin (Figure 8C), both serine proteases revealed loops connecting α-helix and β-strand patterns in their structure with two central cores. All these structural features indicate the reliability of the constructed models, making them potential molecules for molecular docking studies.

After obtaining the theoretical models for our target molecules, molecular docking simulations were carried out to calculate the binding affinities for the molecular complexes inhibitor/protease (Figure 8D,E), and to map the predicted atomic interactions contributing to the stabilisation of these complexes.

The binding affinity value for the ILTI/ trypsin molecular complex (Table 2) was −14.0 kcal mol^−1^. The complex was characterized by 20 atomic interactions, with atomic interactions distances ranging from 3.0 to 4.0 Å. Among them, eight were hydrophobic interactions, 11 were hydrogen bonds, and one saline bond (electrostatic interaction) was mapped (Table 2).

By contrast, as observed in Table 3, the binding affinity value for the molecular complex ILTI/chymotrypsin was −11.3 kcal mol^−1^. This binding affinity is characterized by 11 atomic interactions, three of which are hydrophobic interactions and eight are hydrogen bonds, with atomic interaction distances ranging from 3.1 to 4.0 Å. The binding affinity values for each molecular complex are also shown in the table legends (Table 2 and Table 3). These data support our in vitro results, in which ILTI presented greater potential in inhibiting *Ae. aegypti* trypsin.

## 4. Discussion

Historically, the WHO recommends five classes of insecticides [27]: organochlorines, pyrethroids, carbamates, organophosphates, and spinosyns, all neurotoxic. However, these chemical insecticides have not demonstrated efficiency in controlling vectors. Resistance precedes the use of insecticides, since resistant individuals are a minority in populations. Exposure to insecticides promotes the selection of these naturally resistant individuals, eliminating susceptible ones. Consequently, there is a reduction in the genetic variability of populations, and resistance to chemical insecticides is predominantly established [28]. In the absence of systemic vaccination coverage for the entire population, against arboviruses, alternatives to vector control are urgently needed.

Incorporating ILTI into neonatal larvae revealed a delay in the development of each *Ae. aegypti* larval instar compared to the control group. This effect is related to the amount of inhibitor supplied in the rearing medium. While larvae from the control group reached the pupal stage in 6 days, during the same period, the groups that ingested ILTI (0.06 mgP mL^−1^) still have individuals in the intermediate larval stages after 96 h of treatment.

This delay in development may indicate a lower absorption of nutrients because, to develop regularly, the larvae require a diet that contains many amino acids in addition to glucose and lipids [29]. Similar observations are found when larvae are exposed to nutrient-poor diets. When subjected to diets with a low concentration of lipids and proteins, larvae of this species take up to 15 days longer to reach the pupa stage and have a higher mortality rate [30]. The same developmental delay was found when *Ae. aegypti* larvae were subjected to media containing the trypsin inhibitor present in *Moringa oleifera* (Lam. 1785) flowers (MoFTI) for 72 h [31], demonstrating that PIs frequently present deleterious effects on larval development and death from starvation [32].

Similarly to ILTI, other proteins isolated from plants showed larvicidal activity against *Ae. aegypti*. Among them are lectins isolated from seeds of *M. oleifera* (WSMoL), bark (MuBL), heartwood (MuHL), and leaves (MuLL) of *Myracrondruon urundeuva* (Bentham, 1840). The LC_50_ value of MoFTI for L_1_ is 0.3 mgP mL^−1^ [33], whereas that of *Adenanthera pavonin* (L., 1753) trypsin inhibitor (ApTI) is 0.2 mg mL^−1^ for L_4_ treated for 96 h [15]. These are higher values than those found for ILTI (0.0954 mgP mL^−1^), making ILTI an excellent larvicide for *Ae. aegypti*. Although lectins and peptidase inhibitors act through different pathways in larval metabolism, it is possible to compare the response developed by *Ae. aegypti* larvae subjected to these molecules, since they are macromolecules with protein characteristics and multiple potential for biotechnological use, and they act primarily in the digestive tract of insects [32].

The effect of ILTI on the mass of treated larvae was similar to that found in *Ae. aegypti* when treated with *Bacillus thuringiensi* (BTi). This microbiological agent produces protein crystals toxic to the midgut of larvae. These crystals release prototoxins that form pores in the cell membrane, hindering the assimilation of nutrients and leading to larval death [34]. Other inhibitors from the Kunitz family have similar effects on larval weight and reduced survival, including those isolated in seeds of *Acacia polyphylla* and *Adenanthera pavonina* [35,36]. The *Cassia leiandra* (Benth., 1840) Trypsin Inhibitor (ClTI) proved to be effective in inhibiting digestive proteases in the midgut of *Ae. aegypti* larvae, with a 50% reduction in enzymatic activity at a concentration of 4.65 µM. Furthermore, ClTI delayed larval development, observed after ten days of treatment with a concentration of 15.4 µM, and resulted in a mortality of 44% upon adult emergence [37].

Similar deleterious effects were shown when ILTI was fed to other insects. *Diatraea saccharalis* (Fabricius, 1794) and *Heliothis virescens* (Fabricius, 1777) larvae had 50% lower weight when submitted to diets containing 0.1% and 0.5% ILTI, respectively [38]. Machado et al. [39] demonstrated that ILTI obstructs the expression of trypsins from *Spodoptera frugiperda* (J.E. Smith, 1797), which makes it an effective insecticide for this species. Through analysis of the primary and secondary structures, it was concluded that this inhibitor acts similarly to others in the Kunitz family, blocking the trypsin catalytic site, which prevents the hydrolysis of the substrate and the consequent assimilation of food.

The increase in ILTI concentration in the media supplied to *Ae. aegypti* larvae implies a decrease in tryptic and chymotryptic activity in all larval instars, demonstrating the inhibitor’s action on these enzymes. The decrease in enzymatic activity in both pathways, accompanied by increased mortality and weight loss, indicates that the larva does not present a resistance mechanism to this inhibitor. When supplied to larvae of the lepidopterans *D. saccharalis* and *H. virescens*, ILTI showed similar inhibitory activity, although they are insects from different orders [38].

The trimethyl orthoformate (TMOF), supplied to *Ae. aegypti* larvae, inhibited the insect’s total tryptic activity by 88%, leading to a much smaller mass than the control group. This assay concluded that trypsin is one of the essential enzymes for the complete development of this species [40]. Thus, inhibition of tryptic activity may be one of the primary mechanisms for the larvicidal activity of ILTI.

Interestingly, Macedo et al. [13] reported an absence of activity against bovine chymotrypsin in their in vitro assays. However, it proved effective in inhibiting chymotrypsin present in *Ae. aegypti* larvae. This finding suggests a specificity of the inhibitor for the larval variant of the enzyme, possibly due to structural and conformational differences between chymotrypsin from different species. Such differences may include variations in the amino acid residues that make up the enzyme’s active site, resulting in a distinct three-dimensional conformation that is not efficiently recognized by the trypsin inhibitor. These findings can also be supported by our molecular docking studies, in which a greater binding affinity and a higher number of atomic interactions were predicted for the trypsin/ILTI complexes compared to chymotrypsin/ILTI.

Ex vivo assays revealed that L_4_ fed with ILTI did not exhibit compensatory increases in tryptic activity. This indicates that the inhibitor efficiently suppressed proteinases without inducing immediate metabolic adaptations. The maintained sensitivity to TLCK and PMSF demonstrated that the digestive enzymes remained predominantly serine, confirming the specific action of ILTI on this enzyme group. Notably, ILTI showed greater efficacy (88.4% inhibition) than synthetic inhibitors, suggesting a high affinity for mosquito trypsins. The absence of resistance in the first generation and the stability of the inhibitor in the digestive tract reinforce its potential as a control agent, capable of compromising larval protein nutrition.

These findings position ILTI as a promising alternative for integrated vector management strategies, with effects consistently demonstrated in quantitative assay (BApNA).

ILTI did not alter the acetylcholinesterase activity of *Ae. aegypti* larvae, probably because it is not a target site. Similar results have been reported for MoFTI, which was also unable to reduce the acetylcholinesterase activity of L_4_ of *Ae. aegypti* [33]. These results indicate that this is not one of the mechanisms through which the inhibitor has insecticidal activity. Acetylcholinesterase has systemic importance, acting as a neurotransmitter, and it is a target site for organophosphate insecticides. The toxicity of organophosphates for mammals is related to the non-specificity of this class of compounds [31]. As it does not act on this target, it is possible to suggest that the toxicity of ILTI for humans is also reduced.

ILTI did not interfere with the activity of larval acid phosphatases, but there was a decrease in the activity of alkaline phosphatases when the inhibitor was supplied in higher concentrations. Bti makes use of these phosphatases to promote its toxicity mechanism. This agent has been used to combat larval forms of the mosquito and acts by releasing protein crystals (Cry toxins) that form polymers in cell membranes, causing loss of homeostasis and leading to larval death. Alkaline phosphatases, close to the cell membrane, act as receptors for Cry toxins in Lepidoptera [41]. The silence of the alkaline phosphatase 1 (ALP-1) gene in *Ae. aegypti* larvae resulted in lower Bti toxicity to the insect [42]. When studying this mechanism, Pachecco et al. [43] concluded that ALP-1 is a receptor for the toxins Cry11Aa and Cry4B. When they do not find a receptor, these proteins are eliminated, and the toxic effect is compromised. ILTI used in higher concentrations could reduce the activity of alkaline phosphatases in *Ae. aegypti* larvae, but its concomitant use with Bti must be carried out with caution so that there is no reduction in the effect of Cry toxins because of the reduction of its target site.

## 5. Conclusions

Our results demonstrate that ILTI exerts a potent inhibitory effect on midgut proteases in *Ae. aegypti* larvae, leading to significant developmental delays and increased mortality rates. These findings provide robust evidence of the insecticidal potential of protease inhibitors, underscoring ILTI’s promise as a biotechnological tool for controlling *Ae. aegypti* populations. This study reinforces the relevance of protease inhibitors as viable agents in developing innovative, targeted approaches for mosquito management, offering a promising avenue for reducing disease transmission associated with this vector.

Our findings provide compelling evidence that ILTI exhibits substantial inhibitory activity against midgut proteases in *Ae. aegypti* larvae, manifesting in marked delays in larval development and significantly elevated mortality rates. The suppression of proteolytic activity in the midgut directly impacts essential digestive processes, underscoring the critical role of these enzymes in larval physiology and survival. By targeting these proteases, ILTI disrupts nutrient assimilation, weakening larval fitness and ultimately reducing survival rates.

These results enhance our understanding of the molecular interactions underlying the insecticidal potential of protease inhibitors and suggest that ILTI is a valuable candidate for biotechnological applications aimed at *Ae. aegypti* vector control. This study supports the feasibility of incorporating protease inhibitors as a targeted approach in integrated pest management strategies, focusing on ecological and sustainable alternatives to chemical insecticides. Furthermore, the specificity of ILTI against mosquito proteases highlights its potential to minimise off-target effects, contributing to the development of novel and safer tools for managing mosquito populations.

In summary, this research provides important insights into the mode of action of protease inhibitors as biological insecticidal agents. It paves the way for advanced methods to mitigate *Ae. aegypti*-borne disease transmission, thus addressing a significant public health challenge.

## Figures and Tables

**Table 1:** Structural statistics for the lowest free-energy three-dimensional theoretical models generated in this study for ILTI, trypsin and chymotrypsin.

Predicted Structures	Sequence Length	Stereochemistry (G-Factors)	Most Favored (%)	Outliers (%)	Bad Bonds (%)	Bad Angles (%)	Poor Rotamers (%)	Favored Rotamers (%)
ILTI	178	−0.31	90.4	2.27	1.91	0.84	0.00	98.66
Chymotrypsin	238	−0.36	93.64	3.81	2.22	0.83	0.52	98.96
Trypsin	264	−0.31	93.51	3.44	2.76	0.82	0.00	98.21

**Table 2:** In silico interactions between ILTI/Trypsin (−14.0 kcal mol^−1^) after molecular docking simulations.

Residues	Positions	Atom Names	Distances (Å)	Residues	Positions	Atom Names	Interactions
Trypsin				ILTI			
Lys	64	NZ	3.3	Arg	144	O	HB
Lys	64	CE	3.7	Arg	144	CA	H
Pro	62	O	3.3	Thr	142	O	HB
Tyr	63	O	3.9	Thr	142	O	HB
Ser	61	O	3.4	Thr	142	O	HB
Pro	62	CB	4.0	Phe	136	CZ	H
Lys	64	CG	4.0	Val	143	CB	H
Lys	65	CG	3.8	Val	143	CG1	H
Gln	110	OE1	3.8	Thr	211	OG1	HB
Gln	110	CD	3.7	Thr	211	CB	H
Gln	110	CD	3.8	Thr	211	CG2	H
Gln	110	OE1	4.0	Gln	212	N	HB
Gln	110	OE1	3.3	Gln	212	NE2	HB
Gly	141	N	3.6	Arg	27	NH1	HB
Asp	143	OD1	3.4	Arg	27	NH2	SB
Gln	142	O	3.0	Arg	27	NH2	HB
Gly	141	O	3.8	Arg	27	NH2	HB
Gln	142	O	3.5	Arg	27	NH1	HB
Pro	73	CG	3.7	Tyr	31	CE2	H
Pro	73	CB	3.7	Tyr	31	CE2	H

Å: Angstrom; HB: Hydrogen bond; SB: Saline bond; H: Hydrophobic interaction.

**Table 3:** In silico interactions between ILTI/Chymotrypsin (−11.3 kcal mol^−1^) after molecular docking simulations.

Residues	Positions	Atom Names	Distances (Å)	Residues	Positions	Atom Names	Interactions
Chymotrypsin				ILTI (gi: AFG28551)			
Tyr	51	CE1	3.9	Ala	25	CB	H
Tyr	51	O	3.4	Thr	236	OG1	HB
Tyr	18	OH	3.7	Glu	170	O	HB
Lys	176	NZ	3.5	Ser	40	OG	HB
Gly	26	O	3.9	His	32	NE2	HB
Tyr	80	OH	3.8	Ile	173	N	HB
Tyr	80	OH	3.2	Ile	173	O	HB
Val	85	CG2	3.8	Thr	5	CG2	H
Val	85	CG1	3.9	Ile	6	CG2	H
Pro	83	O	3.1	Gln	66	OE1	HB
Asn	178	ND2	4.0	Ser	169	OG	HB

Å: Angstrom; HB: Hydrogen bond; H: Hydrophobic interaction.

## Data Availability

The original contributions presented in the study are included in the article, further inquiries can be directed to the corresponding author.
